# Knowledge, attitudes, and practices on food safety and hygiene of wet and dry fish handlers in Cox's Bazar, Bangladesh

**DOI:** 10.1002/fsn3.3004

**Published:** 2022-07-27

**Authors:** Mohammad Khairul Alam Sobuj, Ahmad Fazley Rabby, Shafiqur Rahman, Shanur Jahedul Hasan, Shuva Bhowmik, Md. Ariful Islam, Md. Mohidul Islam, Md. Golam Mostofa, Abdullah‐Al Mamun

**Affiliations:** ^1^ Marine Fisheries and Technology Station Bangladesh Fisheries Research Institute Cox's Bazar Bangladesh; ^2^ Department of Fisheries and Marine Science Noakhali Science and Technology University Noakhali Bangladesh; ^3^ Centre for Bioengineering and Nanomedicine, Faculty of Dentistry, Division of Health Sciences University of Otago Dunedin New Zealand; ^4^ Department of Biology University of Louisiana at Lafayette Lafayette Louisiana USA

**Keywords:** attitudes, Bangladesh, fish handlers, food safety, knowledge, practices

## Abstract

This study aimed to provide the very first description of the current scenario of knowledge, attitudes, and practices (KAP) concerning the food safety and hygiene subjects among wet fish handlers (WFHs) and dry fish handlers (DFHs) in Cox's Bazar, Bangladesh. Data collection was performed through the application of face‐to‐face interviews with 234 WFHs and 258 DFHs. The overall score of the correct answer assessed components was 55.95% and 57.05% in WFHs and DFHs, respectively. Among the different knowledge categories, both the respondents showed the highest positive response in the time, temperature, and quality control category and the lowest positive responses noted in the foodborne disease occurrence category. For attitudes, obtained results showed positive attitude with a mean score of 37.82 ± 4.28 and 35.58 ± 5.48 for WFHs and DFHs, respectively. The WFHs gained a mean score of 23.08 ± 4.24 for practices, and the score was 22.78 ± 4.47 in the case of DFHs. A positively significant correlation coefficient (*r*_s_) was observed among fish handlers' KAP of 0.326–0.584. Although the association between the three levels was acceptable, several food safety principles and hygiene practices remained unsatisfactory. These findings highlighted the need for rapid action to enhance food safety and hygiene KAP through an intensive training program to assure the production of safe fisheries products for human consumption.

## Graphical abstract

Both wet and dry fish handlers had adequate levels of knowledge, attitudes, and practices regarding food safety and hygiene. A positively significant correlation coefficient was observed amongst fish handlers’ knowledge, attitudes, and practices of 0.326‐0.584. Need awareness training programme at regular intervals to improve the knowledge level of handlers and to process or handle the fish more safely.

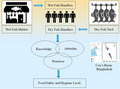

## 1 INTRODUCTION

Fish and fishery products are common agricultural commodities that supply sustenance for humans and animals worldwide (Reksten et al., 2020). The fish production, consumption, and distribution trends in the developing world have expanded dramatically in recent decades (Bogard et al., 2017; Mandal et al., 2021). The global desire for fish and fishery products is booming and gaining popularity due to its potential role in supplying food, nutrition, and livelihoods (FAO, 2020). Fish is an important and readily accessible source of protein for many low‐ to middle‐income countries (LMICs), including Bangladesh, accounting for around 60% of total animal protein demand, with per capita fish intake attaining 62.58 g per day (DoF, 2018). Fisheries are a crucial sector in Bangladesh regarding employment creation, animal protein consumption, foreign exchange earnings, and gross domestic product (GDP) growth. Bangladesh's total fish production has grown substantially by 8–10% yearly (DoF, 2018) as the country has become the 3rd in inland fish, 5th in aquaculture production, and 11th in marine fish production in global ranking in 2018 (FAO, 2018). Fish is a highly perishable item and needs to be maintained in a proper chain of custody immediately after harvesting. Generally, harvested fish come into the landing station and/or fish markets and are then sold as wet fish (fresh, raw, and uncooked fish sold in the market). Additionally, some portions of wet fish enter the drying yard as produced dry fish (processed wet fish having lower moisture content). People who are involved in the loading and unloading/sale of raw fish are called wet fish handlers (WFHs), while people who are involved in the processing and production/sale of dried fish are called dry fish handlers (DFHs) (Faruque et al., 2012; Rao et al., 2005). Bangladesh exports about 1.7% (70,000 mt) of total produced fish in global markets and maintains mostly all required food safety standards, including Hazard Analysis and Critical Control Points (HACCP), Good Manufacturing Practice (GMP), and proper hygiene techniques. However, the remaining bulk amount of fish destined for local consumption is poorly managed. It is assumed that a significant percentage of the wet fish catch loses most of its taste, flavor, and nutritional content when it reaches the customer due to a lack of adequate facilities and knowledge on handling and management (Rasul et al., 2020).

Cox's Bazar is home to Bangladesh's greatest coastline, including the world's longest unbroken natural beach (approximately 125 km) in the southeastern region under the Chattogram division. This division possesses the highest number (76) of fish landing centers, with a total of 237 landing centers in the country (Rahman et al., 2013). The livelihood of the coastal inhabitants in Cox's Bazar combines aquaculture, fish and dried fish, agriculture, salt production, local tourism, trading, and handicrafts (Hena et al., 2005). The Bangladesh Fisheries Development Corporation (BFDC) Fish Landing Centre, a well‐known marine fish landing station, is located here, as anchors of various fishing trawlers and most of the marine catches land here and serve as distribution centers (Miah et al., 2022). The captured fish are transported via various distribution channels from the landing centers to buyer marketplaces before being distributed throughout the country (Rahman et al., 2013). Approximately, 62.0% of the catch is transported within 100.0 km, whereas around 6.0% of the catch is transported >500.0 km (Rahman et al., 2013). These landing centers are critical for supplying high‐quality fresh seafood throughout the country.

Dried fish in Bangladesh is a significant dietary food item (Rakib et al., 2021). Drying is a useful, low‐cost, and potent fish processing approach that reduces the fish body's humidity levels and is used in many areas of the world, particularly in Asian and African countries, to keep fish for a longer duration of time (Rasul et al., 2020). Dried fish is the most frequently consumed kind of fish in some areas of Bangladesh, and it is particularly imperative for low‐income customers. Being a perishable item, fish and fishery products require rapid postharvesting treatment to assure local consumer items' safety and quality. Furthermore, improper storage and management accelerate lipid rancidity in dry fish, resulting in unpleasant aromas, smells, and hazardous hydroperoxide molecules, which are perilous for human consumption (Majumdar et al., 2018). The probable causes of these hazards are traditional drying, use of illegal pesticides (dichlorodiphenyltrichloroethane (DDT), heptachlor, diazinon, etc.) at higher doses, and lack of adequate hygiene, sanitation, packing, preservation, marketing, and water contamination (Rasul et al., 2020). These issues render dried fish unfit for human eating, posing a health hazard to humans that might have negative health consequences.

Moreover, fish is the most commonly eaten animal product in the domestic stage in Bangladesh; however, these fish and fishery products can be a significant source of foodborne illnesses (Belton et al., 2014). Foodborne illnesses are comprised of a diverse range of sicknesses that affect humans in various ways of transmission (EFSA, 2018), especially via food contamination, which is a global public health concern (Luo et al., 2019). The World Health Organization (WHO) revealed that one of every ten people becomes sick each year due to food poisoning which results in considerable morbidity and mortality, and approximately 420,000 deaths occur worldwide because of foodborne illnesses (WHO, 2015). Threats of such foodborne diseases in developing countries like Bangladesh are related to improper handling, inadequate monitoring, decentralized regulatory systems, insufficient protection measures, and the lack of adequate detailed data on food safety concerns (Martins et al., 2012). Certain food items, like fish and fish‐based products, are more vulnerable to foodborne illnesses since they contain pathogens in the guts and outer walls, pollutants, natural toxic substances, and other harmful contaminants (Anal et al., 2020). To meet the ever‐growing fish demand in the country, occupational risks are often overlooked. Infected employees like fish handlers and fish processors are frequently the origins of foodborne viruses, i.e., hepatitis A and diarrhea, as sick fish workers are at high risk of spreading foodborne microbes into the surrounding workplaces. Additionally, the food production process is crucial as foodborne contamination can often occur during bivalve or shellfish processing (Velebit et al., 2019). A lack of time and ambient temperature management knowledge of food products and hazardous contamination can lead to food poisoning outbreaks (Mun, 2020). Furthermore, foodborne illnesses are associated with inadequate sanitary measures, lack of personal hygiene knowledge, unfavorable food preservation temperatures, poor regulatory frameworks, feeble food safety measures, and funding limitations on required tools (Mun, 2020; Odeyemi et al., 2019). In the endeavor to reduce or mitigate the outbreak of endemic foodborne illnesses, sound hygienic food planning and practices are fundamental (Odeyemi et al., 2019).

Knowledge of food safety is important because it can help to prevent the spread of foodborne illnesses (Jianu & Goleţ, 2014). In general, food handlers' attitudes and practices influence their degree of food safety perception (Zanin et al., 2017). Food safety behaviors and practices are influenced by their attitude since a good attitude implies an understanding of safety to allow safe food for everybody (Kwol et al., 2020). The concept of knowledge, attitudes, and practices (KAP) is widely applied to detect food safety concerns at numerous points throughout the value‐added food chain. Although basic research on the KAP of fish farmers and restaurant food handlers has been undertaken previously in the Noakhali district (Hashanuzzaman et al., 2020), no research has addressed food safety and hygiene concerns of both wet and dried fish handlers in Cox's Bazar, Bangladesh, yet. Thus, the major objective of the current research is to evaluate the food safety and hygiene knowledge, attitudes, and practices of wet and dry fish handlers in the Cox's Bazar region of Bangladesh.

## 2 MATERIALS AND METHODS

### 2.1 Study design and sites

**FIGURE 1:**
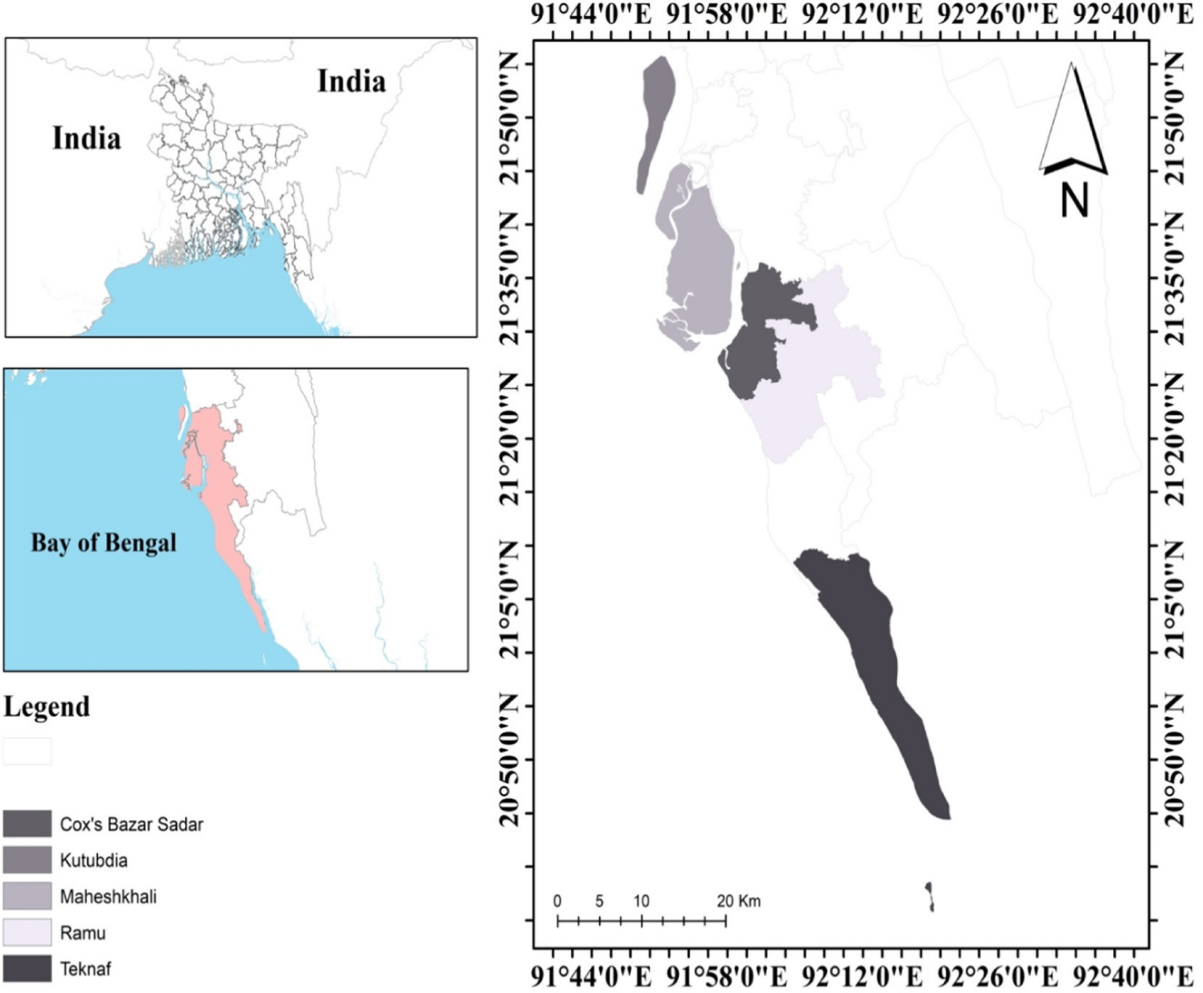
Map showing the data collection sites of the Cox's Bazar region, Bangladesh.

The study was conducted in Cox's Bazar, the main marine dry fish and artisanal fish landing hub of the country. A face‐to‐face cross‐sectional survey using a semi‐structured questionnaire was used to collect data from a total of 492 wet and dry fish handlers from July 2020 to February 2021 (Figure 1). We also followed a simple structural method for identifying the fish markets where wholesaling (selling of bulk amount of fish at a relatively lower price) and/or retailing (selling of small amount of fish directly to the consumer) took place in the Cox's Bazar region. The stratified random sampling was used in 20 fish markets, including one fish landing ground held every day a week. Furthermore, we used a simple structural process to choose dry fish‐producing sites with a processing area. Regardless of scale or number of sales, 34 dry fish‐producing sites where dry fish was produced commercially were randomly chosen.

### 2.2 Study population

This cross‐sectional survey was carried out among 234 WFHs from 20 different fish markets, seven of which were with minimum representation from each market. Furthermore, the research includes 258 DFHs from 34 dry fish production sites, five of which were with minimum representation from each yard. Study populations from both fish markets and dried fish processing sites were chosen at random.

### 2.3 Questionnaire preparation

A group of researchers created the preliminary survey questionnaire form based on an extensive literature review, personal observations, group discussions, and experiences. The questionnaire's framework was primarily founded and developed through the literature review (Bremner, 2002; Ward & Beyens, 2015). Therefore, a questionnaire was planned and developed accordingly (de Vet et al., 2006; Peer & Gamliel, 2011). A questionnaire was created logically, including qualitative and quantitative values of results, so that respondents may react quickly and sequentially. Several issues relevant to wet and dried fish processing have been included in the survey. Each interviewer read the questionnaire and finalized the answer in separate interviews. The whole questionnaire was organized and separated into five sections: section 1: demographic information (such as gender, age, family size, religion, educational level, employment status, electricity facilities, toilet facilities, and monthly income); section 2: information about employees' work satisfaction and the COVID‐19 pandemic situation; section 3: knowledge about food safety and hygiene; section 4: attitudes about food safety and hygiene; and section 5: food safety and hygiene practices of wet and dry fish handlers.

To understand the level of KAP and the interaction between different responses of WFHs and DFHs, scoring methods were used. Respondents were asked to pick the best answer from three options (agree, neutral, and disagree) in multiple‐choice questions. For multiple‐choice questions, greater scores were given to responses that followed the most food handling and safety guidelines. Participants' responses were graded on a three‐point Likert scale, with three points awarded for agree, two points awarded for neutral, and one point awarded for disagree in the case of positively formed questions, and points were awarded in the opposite sequence for negatively formed questions. Finally, based on the scores (*x*), participants were deemed to have insufficient knowledge (*x* ≤ 30), adequate knowledge (30 < *x* ≤ 60), and good knowledge (60 < *x* ≤ 90) of food handling and safety (Hashanuzzaman et al., 2020).

### 2.4 Piloting the study

The questionnaire was piloted, emphasizing its ability to be understood, relation to the intended subjects, reliability in presenting relevant knowledge, and the level to which the questions are perceived and accepted by different people. A total of 28 WFHs and 30 DFHs from three fish markets and 5 dry fish processing sites were chosen purposively in a parallel setting for the pretesting/piloting. After piloting, the questionnaire was tested using Cronbach's alpha test, having a reliability coefficient of 0.79, showing the questionnaire's accuracy and suitability for the study (Santos, 1999). The survey relied entirely on direct observations, ensuring that data on actual activity could be gathered (da Cunha et al., 2019). Finally, the KAP survey questionnaire was initially composed in English and then converted into the local Bengali language to make the procedure go more smoothly.

### 2.5 Data collection

Face‐to‐face interviews with WFHs and DFHs were conducted at their respective fish markets and dry fish processing sites. The survey was entirely voluntary, private, and anonymous. The participants were also informed of the study's voluntary character, and they were encouraged to opt‐out if they so desired. Wet and dried fish handlers volunteered to participate in the study after being briefed about the topic and objectives of the survey and assured of its privacy. The station chief of the Marine Fisheries and Technology Station, Bangladesh Fisheries Research Institute, Cox's Bazar, gave his prior approval to conduct this survey. The research's goal and concept were also clearly and briefly addressed with the individual WFHs and the owner and/or supervisor of the dry fish processing sites. To obtain reliable data, a friendly relationship was initially created with the employees. The owner or supervisor was also asked a few essential questions before speaking with their employees at a remote position.

### 2.6 Statistical analysis

The results of the experiment were evaluated using a standard statistical method. Data were analyzed using SPSS (Statistical Package for the social sciences) software (IBM Co., Chicago, IL) version 21. Different variables were compared using the analysis of variance (ANOVA) and Duncan's multiple‐range technique. Differences were considered significant at *p* < .05.

## 3 RESULTS AND DISCUSSION

### 3.1 Demographic characteristics of respondents

Demographic characteristics, including gender, age, family size, religion, educational level, employment status, working experience, electricity facilities, toilet facilities, and monthly income of WFHs (*n* = 234) and DFHs (*n* = 258) in the Cox's Bazar region, are presented in Table 1. Males represent the majority of WFHs (98.7%), whereas females (71.3%) represent the majority of DFHs. WFHs (46.2%) were mostly between the ages of 41 and 60, whereas DFHs (37.6%) were mostly under the age of 20. There was a majority of Muslim participants (92.3%) with no formal education (62.1%) in the case of DFHs. Most of the WFHs (79.9%) were engaged in full‐time jobs, whereas the situation was the opposite in the case of DFHs (40.3%). This is because the fish drying process largely depends on the season and availability of fish species. Approximately 39.8% of WFHs have been involved in their profession for about 20 years or more. The majority of WFHs (56.4%) earned between USD 100 and 180 per month, whereas the majority of DFHs (53.1%) earned less than USD 100 per month.

**TABLE 1:** Demographic characteristics of WFHs (*n* = 234) and DFHs (*n* = 258) in Cox's Bazar, Bangladesh

Category	Sub category	Wet fish handlers *n* (%)	Dry fish handlers *n* (%)
Gender	Female	3 (1.3)	184 (71.3)
	Male	231 (98.7)	74 (28.7)
Age	≤20 years	26 (11.1)	97 (37.6)
	21–40 years	56 (23.9)	74 (28.7)
	41–60 years	108 (46.2)	65 (25.2)
	Above 60 years	44 (18.8)	22 (8.5)
Family size	Small (≤4)	36 (15.4)	58 (22.5)
	Medium (5–6)	51 (21.8)	89 (34.5)
	Large (>6)	147 (62.8)	111 (43.0)
Religion	Muslim	194 (82.9)	238 (92.3)
	Hindu	31 (13.2)	14 (5.4)
	Others	9 (3.9)	6 (2.3)
Educational level	Illiterate/Can sign	111 (47.5)	160 (62.1)
	Up to primary	92 (39.3)	71 (27.5)
	Up to secondary	19 (8.1)	22 (8.5)
	Above secondary	12 (5.1)	5 (1.9)
Employment status as fish handler	Full time	187 (79.9)	104 (40.3)
	Part time	47 (20.1)	154 (59.7)
Working experience	≤2 years	22 (9.4)	31 (12.0)
	3–10 years	56 (23.9)	75 (29.1)
	11–20 years	63 (26.9)	89 (34.5)
	Above 20 years	93 (39.8)	63 (24.4)
Electricity facilities	No electricity/No solar	17 (7.3)	21 (8.1)
	Solar	48 (20.5)	87 (33.7)
	Electricity	169 (72.2)	150 (58.2)
Toilet facilities	Open space	31 (13.3)	49 (19.0)
	Nonsanitary	78 (33.3)	86 (33.3)
	Sanitary without water seal	83 (35.5)	72 (27.9)
	Sanitary with water seal	42 (17.9)	51 (19.8)
Monthly income in USD^a^	Below 100	38 (16.2)	137 (53.1)
	100–180	132 (56.4)	80 (31.0)
	Above 180	64 (27.4)	41 (15.9)

a Exchange rate 1 USD = 85.0 Bangladeshi Taka (BDT).

### 3.2 Employees' work satisfaction and the COVID‐19 pandemic situation

Employees' satisfaction with the institution's COVID‐19 statements has a beneficial effect on employees' willingness to exert any effort in their jobs (Vo‐Thanh et al., 2020). Thus, the satisfaction level of fish handlers with their working circumstances, job load, work relations, and other people's reactions to them in the COVID‐19 situation was assessed through the survey (Table 2). In general, all of the fish handlers assured us that they were aware of the organization's standards and rules and always tried to follow the rules. The consequences of the COVID‐19 pandemic are not always health related but also have a social and financial impact since many small‐ and medium‐sized enterprises in Bangladesh have been directly impacted (Bhuiyan et al., 2020). In our study, we found that 55.1% of WFHs' and 72.5% of DFHs' authorities were indifferent to managing COVID‐19 at their institutions. In the case of maintaining social distancing, dry fish authorities were more aware compared to the wet fish authorities, as confirmed by the responses.

**TABLE 2:** Assessment of employees' work satisfaction and the COVID‐19 pandemic situation

Statements	Responses of fish handlers^a^ *n* (%)					
	Wet fish handlers			Dry fish handlers		
	Agree *n* (%)	Neutral *n* (%)	Disagree *n* (%)	Agree *n* (%)	Neutral *n* (%)	Disagree *n* (%)
Would you prefer the same occupation if you had the option?	107 (45.7)	46 (19.7)	81 (34.6)	143 (55.4)	40 (15.5)	75 (29.1)
Did you feel free to share your problems with your coworkers?	198 (84.6)	0 (0)	36 (15.4)	228 (88.4)	0 (0)	30 (11.6)
Did you always follow the organization's standards and rules?	234 (100)	0 (0)	0 (0)	258 (100)	0 (0)	0 (0)
Will you quit this job if anyone gave you anything better elsewhere?	74 (32)	19 (8)	141 (60)	79 (30.6)	11 (4.3)	168 (65.1)
Is the workload sufficient for you?	211 (90.2)	0 (0)	23 (9.8)	209 (81.0)	0 (0)	49 (19.0)
Does the fishy smell bother you?	0 (0)	0 (0)	234 (100)	0 (0)	0 (0)	258 (100)
Does the worker respect each other at the workplace?	173 (73.9)	32 (13.7)	29 (12.4)	217 (84.1)	15 (5.8)	26 (10.1)
Does the authority always concern about workplace hazards and high‐risk work?	97 (41.5)	80 (34.2)	57 (24.3)	130 (50.4)	52 (20.2)	76 (29.4)
Does the authority provide all the necessary steps to manage COVID‐19?	24 (10.3)	81 (34.6)	129 (55.1)	44 (17.1)	27 (10.4)	187 (72.5)
Does the workplace in such a way that social distance can be maintained?	39 (16.7)	0 (0)	195 (83.3)	206 (79.8)	0 (0)	52 (20.2)
Does the authority take immediate action during COVID‐19 suspected cases in the workplace?	151 (64.5)	22 (9.4)	61 (26.1)	175 (67.8)	15 (5.8)	68 (26.4)

^a^ *n* (%) – number of respondents (percentage of respondents).

### 3.3 Food safety knowledge of respondents

The estimation of the correct answers in the assessed components (30 questions) was used to determine the overall food safety knowledge score. The overall score of the correct answers in the assessed components (personal hygiene, cross‐contamination and hygiene, time, temperature, and quality control, and foodborne disease occurrence) was 55.95% and 57.05%, respectively, in the case of WFHs and DFHs (Table 3).

**TABLE 3:** Food safety and hygiene knowledge of wet fish handlers (WFHs) (*n* = 234) and dry fish handlers (DFHs) (*n* = 258) in Cox's Bazar, Bangladesh

Category	Statements	Responses of fish handlers^a^ *n* (%)									
		Wet fish handlers (*n* = 234)					Dry fish handlers (*n* = 258)				
		Agree *n* (%)	Neutral *n* (%)	Disagree *n* (%)	χ^2^	*p*‐Value	Agree *n* (%)	Neutral *n* (%)	Disagree *n* (%)	χ^2^	*p*‐Value
Personal hygiene	Washing hands earlier at work reduces the risk of external contamination	**25 (10.7)**	130 (55.6)	79 (33.7)	5.963	.051	31 (12.0)	183 (70.9)	44 (17.1)	97.182	.000*
	Several types of hazards cause contamination	**19 (8.1)**	148 (63.3)	67 (28.6)	7.575	.023*	68 (26.4)	127 (49.2)	63 (24.4)	230.054	.000*
	Using protective gloves while handling fish decreases the chance of fish contamination	**26 (11.1)**	101 (43.2)	107 (45.7)	3.606	.165	34 (13.2)	159 (61.6)	65 (25.2)	111.648	.000*
	Personal hygiene can prevent food contamination	**81 (34.7)**	42 (17.9)	111 (47.4)	3.367	.186	143 (55.4)	80 (31.0)	35 (13.6)	83.444	.000*
	Taste or distributing any food with unprotected hands is harmful to health	**193 (82.5)**	41 (17.5)	0 (0)	14.305	.000*	212 (82.2)	32 (12.4)	14 (5.4)	22.514	.000*
	While coughing or sneezing, it is necessary to use protective measures to cover your mouth	**140 (59.9)**	66 (28.2)	28 (11.9)	22.358	.000*	165 (63.9)	41 (15.9)	52 (20.2)	58.483	.000*
	Fish handlers with wounds or symptoms of diseases/infections should not work on the activities	**197 (84.2)**	37 (15.8)	0 (0)	16.180	.000*	219 (84.9)	39 (15.1)	0 (0)	18.478	.000*
	It is essential to ensure social distance at the workplace in this COVID‐19 pandemic	**202 (86.3)**	24 (10.3)	8 (3.4)	85.851	.000*	198 (76.7)	18 (7.0)	42 (16.3)	31.443	.000*
Cross‐contamination and hygiene	A clean working environment is essential in the prevention of contamination	**189 (80.8)**	45 (19.2)	0 (0)	12.764	.000*	164 (63.6)	71 (27.5)	23 (8.9)	59.473	.000*
	Cleaning all fish touch surfaces with water and soap before using a sanitizer is important	**137 (58.6)**	38 (16.2)	59 (25.2)	9.014	.011*	141 (54.7)	40 (15.5)	77 (29.8)	86.099	.000*
	Using different platters and instruments to process different types of fish is essential	30 (12.8)	37 (15.8)	**167 (71.4)**	1.219	.544	68 (26.4)	37 (14.3)	153 (59.3)	233.425	.000*
	Washing the knife used to cut wet fish with water or sanitizer before using it is important	**154 (65.8)**	43 (18.4)	37 (15.8)	16.179	.000*	123 (47.7)	84 (32.5)	51 (19.8)	113.884	.000*
	Washing fish contact surfaces with water and sanitizer every day is important	**208 (88.9)**	26 (11.1)	0 (0)	24.312	.000*	189 (73.3)	11 (4.2)	58 (22.5)	37.881	.000*
	Wet fish might come in contact with processed/dry fish	110 (47.0)	53 (22.7)	**71 (30.3)**	6.977	.031*	42 (16.3)	19 (7.4)	197 (76.3)	198.641	.000*
Time, temperature, and quality control	To maintain the quality of the fish, it needs to be kept in a refrigerator under 40°F	**164 (70.1)**	42 (17.9)	28 (12.0)	22.358	.000*	185 (71.7)	42 (16.3)	31 (12.0)	40.943	.000*
	It is best to store wet fish in a refrigerator for 2–3 days	**89 (38.0)**	39 (16.7)	106 (45.3)	3.670	.160	88 (34.1)	56 (21.7)	114 (44.2)	200.447	.000*
	Lean fish will store longer than oily fish	15 (6.4)	40 (17.1)	**179 (76.5)**	0.934	.627	24 (9.3)	30 (11.6)	204 (79.1)	169.812	.000*
	Whole fish will store better than steaks and fillets	**188 (80.3)**	29 (12.4)	17 (7.3)	38.791	.000*	178 (69.0)	33 (12.8)	47 (18.2)	46.634	.000*
	Preserving the fish while it is as fresh as possible	**208 (88.9)**	16 (6.8)	10 (4.3)	68.073	.000*	184 (71.3)	17 (6.6)	57 (22.1)	41.730	.000*
	Freezing destroys the bacteria that could cause food poisoning	161 (68.8)	29 (12.4)	**44 (18.8)**	13.123	.001*	172 (66.7)	21 (8.1)	65 (25.2)	51.880	.000*
	Ice and refrigeration in general, making the possible extension of fish shelf life	**170 (72.6)**	51 (21.8)	13 (5.6)	51.662	.000*	216 (83.7)	9 (3.5)	33 (12.8)	20.176	.000*
	Fish might be refrozen again that have been thawed	64 (27.4)	37 (15.8)	**133 (56.8)**	2.308	.315	71 (27.5)	45 (17.5)	142 (55.0)	244.312	.000*
	Properly preparing fish for storage will allow it to be stored for a longer period and maintain its quality	**227 (97.0)**	7 (3.0)	0 (0)	98.549	.000*	213 (82.6)	13 (5.0)	32 (12.4)	21.912	.000*
	Contaminated fishes always have some variation in color, texture, odor, or taste	**226 (96.6)**	0 (0)	8 (3.4)	85.851	.000*	238 (92.2)	0 (0)	20 (7.8)	8.719	.003*
	Improper fish storage can threaten consumers' health	**163 (69.7)**	36 (15.4)	35 (14.9)	17.279	.000*	206 (79.8)	29 (11.3)	23 (8.9)	26.192	.000*
Foodborne disease occurrence	The foodborne disease affects all, including infants, teenagers, pregnant women, and the elderly	**108 (46.2)**	30 (12.8)	96 (41.0)	4.369	.113	167 (64.7)	74 (28.7)	17 (6.6)	56.540	.000*
	Good hygiene practices can help to avoid and manage foodborne diseases	**177 (75.6)**	48 (20.5)	9 (3.9)	75.974	.000*	184 (71.3)	44 (17.1)	30 (11.6)	41.730	.000*
	Foodborne diseases have no impact on your health and also the economy.	54 (23.1)	81 (34.6)	**99 (42.3)**	4.144	.126	59 (22.9)	104 (40.3)	95 (36.8)	195.247	.000*
	Hepatitis A virus is among the foodborne pathogens	**62 (26.5)**	117 (50.0)	55 (23.5)	9.890	.007*	139 (53.9)	96 (37.2)	23 (8.9)	88.831	.000*
	*Vibrio parahaemolyticus* is most commonly found in shellfish	**35 (15.0)**	174 (74.4)	25 (10.6)	25.406	.000*	12 (4.6)	212 (82.2)	34 (13.2)	43.545	.000*
Total correct answer percentage		55.95%^b^					57.05%^b^				
Total mean score of knowledge		54.42 ± 10.81^c^					59.38 ± 9.77^c^				

*Note*: Fish handlers' responses in bold are the correct answer.

^a^ *n* (%) – number of respondents (percentage of respondents).

^b^ Total correct answer percentage of the responders.

^c^ Total (mean ± standard deviation) score of the respondents.

* Statistically significant (*p* < .05).

#### 3.3.1 Personal hygiene

**FIGURE 2:**
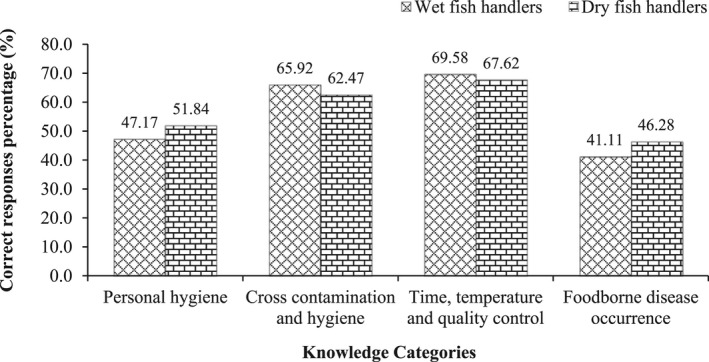
Wet and dry fish handlers correct responses (%) according to different knowledge categories.

A general overview of WFHs' and DFHs' knowledge about food safety is depicted in Figure 2. Only about half of the respondents (47.17% of WFHs and 51.84% of DFHs) knew the answers to the questions. Personal hygiene questionnaires demonstrated that in both groups of participants, hand washing was not as often as predicted. This might suggest inefficiency or a lack of training in hand washing. On the other hand, as wet or dry fish will not be served or consumed directly (need to cook), the handlers might be indifferent to hand washing practices, although to avoid the transmission of harmful germs from workers to food, correct and appropriate hand washing is crucial (Montville et al., 2001). Other academics have also said that proper hygiene and sanitation are not adequately observed in Bangladesh when it comes to the drying of fish (Hasan et al., 2016; Nowsad, 2007). Parallel results have been found in other studies demonstrating a lack of hand washing practices (Osaili et al., 2017). However, according to some studies, hand hygiene is more essential than washing and disinfecting food contact surfaces when it comes to disease management (Todd et al., 2009). More correct responses were obtained to questions related to distributing foods with unprotected hands, taking a break during a disease syndrome, and maintaining social distance. Almost identical replies were found regarding handlers' awareness of personal hygiene in the cases of other researchers (Al‐Shabib et al., 2016; Hashanuzzaman et al., 2020).

#### 3.3.2 Cross‐contamination and hygiene

About 65.92% of WFHs and 62.47% of DFHs delivered correct responses to cross‐contamination and hygiene‐related questions. Here, our current investigation demonstrated that certain fish handlers possessed the necessary expertise to prevent cross‐contamination from occurring. Additionally, several participants provided correct responses to the negatively formed questions, which suggests that they had honestly given their full attention to reading the questions (Table 3). Approximately 76.3% of DFHs knew that dry or processed fish should not come in contact with wet fish. Furthermore, the remaining erroneous respondents may represent a concern since they conduct their tasks incorrectly. As a result of these operations being conducted improperly, foodborne illness risks are high. This might also suggest inefficiency or a lack of cross‐contamination and hygiene training. Previous researchers have found that handlers lack fundamental understanding of cross‐contamination and sanitization procedures, which is similar to our present findings (Al‐Kandari et al., 2019; Osaili et al., 2017), although a greater understanding of cross‐contamination prevention among hospital food service workers was observed in previous research (Buccheri et al., 2010). Furthermore, food handler training and supervision, as well as the use of conventional sanitation methods and insufficient sanitization of food‐contact surfaces can prompt cross‐contamination (Sneed et al., 2004).

#### 3.3.3 Time, temperature, and quality control

The overall knowledge of time, temperature, and quality control of WFHs (69.58%) and DFHs (67.62%) was found to be fair compared to other categories (Figure 2). About 70.1% of WFHs and 71.7% of DFHs responded correctly when asked about the freezing temperature of the fish. Furthermore, in the case of negatively formed questions, several participants provided correct answers, which suggests that they had honestly given their full attention to reading the questions (Table 3). For the consumer health‐related questions, 69.7% of WFHs and 79.8% of DFHs provided correct answers. Other researchers stated that consumers have poor knowledge of time and temperature management in the case of ready‐to‐eat foods (Al‐Kandari et al., 2019; Sani & Siow, 2014). The relatively fair responses obtained from our study could be attributed to the experience of both fish handlers as well as the random use of the refrigerator for preserving the fish. Furthermore, the other inaccurate respondents might be a worry since they do their duties erroneously. According to the World Health Organization, foodborne outbreaks can occur on account of a variety of causes, one of which is the misuse of time and temperature controls when food is being handled (WHO, 2017). These outcomes highlight the relevance of food safety training, in which food handlers' understanding is improved via education, allowing them to conduct sanitary procedures more effectively (Kunadu et al., 2016).

#### 3.3.4 Foodborne disease occurrence

According to Figure 2, 41.11% of WFHs and 46.28% of DFHs were found to have foodborne disease occurrence knowledge, which is the minimum knowledge score obtained in the current study. Only about 26.5% of WFHs and 53.9% of DFHs knew about the hepatitis A virus and its role in foodborne disease occurrence. Although the hepatitis A virus is considered a major causative agent of foodborne illness issues, it is linked to contaminated water or food, poor sanitation practices, and inadequate personal hygiene (WHO, 2015). Surprisingly, only about 15% of WFHs and 4.6% of DFHs were familiar with *Vibrio parahaemolyticus*, which is most commonly found in shellfish organisms. The majority of them remained neutral or kept quiet in this regard, which indicates that they do not know anything about this matter. Other researchers have reported that the respondent's knowledge of foodborne diseases is inadequate, and our results corroborate those findings (Al‐Kandari et al., 2019; Al‐Shabib et al., 2016). The results suggest that there is a necessity to enhance food safety education among fish handlers, principally in terms of foodborne disease occurrence. Consequently, more knowledge does not necessarily result in improvements in handling practices (Ansari‐Lari et al., 2010). There is common agreement that handlers must be knowledgeable about food safety and be able to apply that knowledge during food handling (Mortlock et al., 1999). However, inadequate understanding may reduce one's level of consciousness when it comes to handling, leading to the practice of a deceptive sense of safety (Martins et al., 2012). Thus, fish handlers' attitudes regarding food safety concerns and their ability to affect behaviors must be better understood to transfer knowledge accurately into these areas of food handling practice (Ko, 2013).

### 3.4 Food safety attitudes of respondents

Attitude is considered a significant component that can affect food safety behavior and practice among food handlers, minimizing the prevalence of foodborne diseases and associated health risks (Al‐Shabib et al., 2016). Concerning food handling, attitude is crucial as knowledge and practices are intertwined with it; staff with a positive attitude will be more likely to put their training into practice, and the reverse is true as well (Zanin et al., 2017). Attitudes of the WFHs were obtained with total mean scores of 37.82 ± 4.28 and a mean score of 2.52 (Table 4). Almost all of the WFHs (94.9%) showed a willingness to modify their behavior, when it was wrongly directed. On the other hand, <50% of WFHs showed a positive attitude towards maintaining a high degree of personal hygiene at their workplace, which is similar to the personal hygiene findings of the knowledge categories. Around 70.5% and 79.1% of the WFHs agreed with the following, respectively: the importance of regularly reviewing the chillers' and freezers' temperature settings and supervising the discarding of spoilage or contaminated fish immediately. These results coincide with those of Al‐Shabib et al. (2016), where about 59.2% of respondents strongly agreed that regularly checking the temperature settings of chillers or freezers is necessary. According to Abdul Mutalib et al. (Abdul‐Mutalib et al., 2012), knowledgeable food handlers might result in positive attitudes and even good patterns of behavior. For this reason, administrators must assist in making training a prerequisite for all fish handlers and must comprehend their employees' behavior and how it correlates with their beliefs and knowledge level; thus, the program will be more successful (Sani & Siow, 2014). The statement related to fish handlers who have cuts, scratches, or injuries to their hands being forbidden from touching or handling fish was approved by 61.5% of handlers. However, Zanin et al. (2015) and Al‐Kandari et al. (2019) reported that 85% and 70.1% of their food staff were aware of the risks of handling meals with wounds on their hands or fingers, respectively. According to the FDA (2018), handlers with infections or injuries on their hands should quit working promptly and leave their workplace instantly to prevent foodborne illnesses.

**TABLE 4:** Food safety and hygiene attitude of wet fish handlers (WFHs) (*n* = 234) in Cox's Bazar

Statements	Responses of wet fish handlers^a^ *n* (%)					
	Agree *n* (%)	Neutral *n* (%)	Disagree *n* (%)	Mean ± *SD*	χ^2^	*p*‐Value
Safe fish handling to avoid contamination and increasing shelf life is part of my job responsibilities	164 (70.1)	27 (11.5)	43 (18.4)	2.48 ± 0.19	13.499	.001*
Personal hygiene can help to avoid foodborne illnesses	122 (52.1)	48 (20.5)	64 (27.4)	2.55 ± 0.33	8.072	.018*
Learning more about food safety through training courses is important	208 (88.9)	0 (0)	26 (11.1)	2.79 ± 0.16	24.312	.000*
When I know my fish handling behaviors are wrong, I am willing to modify them	222 (94.9)	0 (0)	12 (5.1)	2.81 ± 0.13	56.221	.000*
We should not rub our hands on our face, hair, or anything else while handling fish	158 (67.5)	19 (8.1)	57 (24.4)	2.23 ± 0.46	9.437	.009*
It is vital to maintain a high degree of personal hygiene at the workplace	104 (44.5)	81 (34.6)	49 (20.9)	2.38 ± 0.25	11.474	.003*
Wearing masks, gloves, caps, and appropriate footwear is an effective precaution to minimize the chance of contamination	67 (28.6)	129 (55.1)	38 (16.3)	1.92 ± 0.41	15.675	.000*
Foodborne illness is more harmful to vulnerable populations (i.e., children, older people, and pregnant women)	191 (81.6)	30 (12.8)	13 (5.6)	2.77 ± 0.20	20.665	.000*
It is important to review the temperature settings of chillers and freezers on a regular basis	165 (70.5)	8 (3.4)	61 (26.1)	2.60 ± 0.27	8.619	.013*
Careful supervision is needed for spoilage or contaminated fish, which should be discarded immediately	185 (79.1)	10 (4.3)	39 (16.6)	2.36 ± 0.38	15.195	.001*
Handlers who have cuts, scratches, or injuries to their hands are forbidden from touching or handling fish	144 (61.5)	22 (9.4)	68 (29.1)	2.49 ± 0.33	7.419	.024*
Proper fish preservation is critical for food safety	201 (85.9)	33 (14.1)	0 (0)	2.83 ± 0.12	18.510	.000*
Ice produced in contaminated water can cause disease	126 (53.9)	37 (15.8)	71 (30.3)	2.28 ± 0.53	6.977	.031*
Fish stored at room temperature may become contaminated	224 (95.7)	10 (4.3)	0 (0)	2.85 ± 0.13	68.073	.000*
Washing wet fish with contaminated water is harmful to health	119 (50.8)	24 (10.3)	91 (38.9)	2.48 ± 0.39	4.776	.092
Total mean score of attitudes of wet fish handlers (WFHs)				37.82 ± 4.28^b^		
Mean of scores				2.52		

^a^ *n* (%) – number of respondents (percentage of respondents).

^b^ Total (mean ± standard deviation) score of the respondents.

* Statistically significant (*p* < .05).

According to the KAP model, handlers possessing adequate food safety knowledge further exhibit favorable, positive attitudes toward food safety procedures, which include personal hygiene, cross‐contamination, and primary disease prevention practices (Asmawi et al., 2018). Table 5 represents the food safety and hygiene attitude of DFHs with a total mean score of 35.58 ± 5.48 and a mean score of 2.37 in the Cox's Bazar region. A very minor percent (16.3%) of DFHs agreed on the importance of hand washing earlier in handling the fish, which also reflects the personal hygiene findings of the knowledge categories. Approximately 57% of the DFHs agreed that workers who have scrapes or injuries to their hands or fingers should not touch fish. However, Hashanuzzaman et al. (2020) and Al‐Kandari et al. (2019) reported that 84% and 70.1% of their food service operators were conscious of the threat of handling foods with cuts on their hands or fingers, respectively, although poor positive responses were obtained in the case of attitudes toward glove changing (28.7%) and bacterial contamination of fish (41.9%). Thongpalad et al. (2019) indicated that handlers appeared to know a lot about food safety, but attitudes are just as essential as knowledge for getting the job done at the field level. Thus, the support and inspiration of workers at the manager's site could be a strategy for increasing knowledge and adopting positive attitudes, which could subsequently help to reduce the number of foodborne disease outbreaks at the dry fish processing sites (Todd et al., 2009).

**TABLE 5:** Food safety and hygiene attitude of dry fish handlers (DFHs) (*n* = 258) in Cox's Bazar

Statements	Responses of dry fish handlers^a^ *n* (%)					
	Agree *n* (%)	Neutral *n* (%)	Disagree *n* (%)	Mean ± *SD*	χ^2^	*p*‐Value
Safe fish preservation to ensure its decent quality is a crucial aspect of my job responsibility	194 (75.2)	25 (9.7)	39 (15.1)	2.39 ± 0.48	34.230	.000*
Prior to beginning work, the work area must be swept	214 (82.9)	0 (0)	44 (17.1)	2.68 ± 0.20	21.334	.000*
Washing hands before handling fish is important	42 (16.3)	177 (68.6)	39 (15.1)	1.94 ± 0.61	129.845	.000*
Workers who have scrapes or injuries to their hands or fingers should not touch fish	149 (57.7)	58 (22.5)	51 (19.8)	2.35 ± 0.43	75.906	.000*
Improper storage of dry fish can be hazardous to health	204 (79.1)	19 (7.3)	35 (13.6)	2.48 ± 0.42	27.466	.009*
Dry fish must be preserved in a dry and clean place	239 (92.6)	10 (3.9)	9 (3.5)	2.56 ± 0.33	8.249	.016*
For handling wet and dry fish, handlers must change gloves	74 (28.7)	128 (49.6)	56 (21.7)	2.09 ± 0.44	258.0	.000*
In direct sun drying, the possibility of contamination with dust and sand, and infestation with insects, their eggs, and larvae are high	245 (95.0)	13 (5.0)	0 (0)	2.81 ± 0.15	5.506	.019*
Packaging is done to make sure that the product is safe for the customers rather than to attract the customers	118 (45.7)	44 (17.1)	96 (37.2)	2.18 ± 0.32	123.106	.000*
Use of insecticides in fish drying process delays insect infestation but it is very harmful to human health	201 (77.9)	22 (8.5)	35 (13.6)	2.60 ± 0.28	29.425	.000*
Before the drying process, quality assessment of wet fishes should be done	48 (18.6)	187 (72.5)	23 (8.9)	1.85 ± 0.60	148.567	.000*
Spoiled or contaminated fish should be discarded from the drying process	199 (77.1)	27 (10.5)	32 (12.4)	2.52 ± 0.29	30.763	.000*
Flies, insects, or rodents can cause bacterial contamination of fish	108 (41.9)	94 (36.4)	56 (21.7)	2.03 ± 0.41	144.112	.000*
Wet and dry fishes should be kept separate	238 (92.2)	17 (6.6)	3 (1.2)	2.67 ± 0.24	8.719	.013*
Wet fish should be washed with clean running water before drying	185 (71.7)	10 (3.9)	63 (24.4)	2.43 ± 0.28	40.943	.000*
Total mean score of attitudes of dry fish handlers (DFHs)				35.58 ± 5.48^b^		
Mean of scores				2.37		

^a^ *n* (%) – number of respondents (percentage of respondents).

^b^ Total (mean ± standard deviation) score of the respondents.

* Statistically significant (*p* < .05).

### 3.5 Food safety practices of respondents

Personal hygiene practices are considered crucial for the processing of food items that are trustworthy for customers. Table 6 displays the WFHs' reactions to 10 different types of practices having total mean scores of 23.08 ± 4.24 and a mean score of 2.31. The results indicate that a very minor percentage exhibited hand washing practices before handling money (27.8%), and before and after rubbing their nose or scratching their body (20.5%), although about 85% and 65% of WFHs and DFHs, respectively, agreed that they wipe their hands after the end of the day's work. These hand washing practices coincide with those found by Hashanuzzaman et al. (2020), who stated that about 88% of the fish farmers performed hand washing practices before eating, and another 60% showed hand washing practices after using the toilet. Soon and Baines (2012) also found that around 60% of farmworkers wiped their hands following sneezing and coughing frequently. Only one‐third of WFHs used protective gloves while handling fish. Sani and Siow (2014) and Çakıroğlu and Uçar (2008) reported that 46.6% and 82.9% of their workers, respectively, wore caps, masks, and gloves when handling food. Poor hand hygiene is a crucial risk factor in the incidence of food contamination, and inappropriate food handling is a primary source of foodborne illnesses (Codex Alimentarius Commission, 2003). Fish handlers must therefore carefully wash their hands at all stages of fish processing, especially before handling fish, after contacting contaminated objects, before and after taking meals or handling money, after using the washroom, and so on. Nonetheless, due to a poor understanding of fish handlers' food safety, they are in the habit of using carcinogenic and hazardous chemicals such as formalin in the fish preservation process (Goon et al., 2014). In addition, good food safety knowledge aids fish handlers in alternative fish processing and preservation technologies, which are essential for prolonging the shelf life of fish and ensuring safe food for public health (Mohan et al., 2018).

**TABLE 6:** Food safety and hygiene practices of wet fish handlers (WFHs) (*n* = 234) in Cox's Bazar

Statements	Responses of wet fish handlers^a^ *n* (%)					
	Agree *n* (%)	Neutral *n* (%)	Disagree *n* (%)	Mean ± *SD*	χ^2^	*p*‐Value
Do you wash your hands properly before handling money?	65 (27.8)	0 (0)	169 (72.2)	2.18 ± 0.44	1.169	0.280
Do you cover your mouth with tissue paper while coughing or sneezing?	29 (12.4)	61 (26.1)	144 (61.5)	1.86 ± 0.56	1.899	0.387
Do you wipe your hands after the end of the day's work?	198 (84.6)	0 (0)	36 (15.4)	2.65 ± 0.37	16.714	0.000*
Do you wash your hands before and after rubbing your nose or scratching your body?	48 (20.5)	30 (12.8)	156 (66.7)	2.28 ± 0.24	1.519	0.468
Do you use protective gloves while handling wet fish?	78 (33.3)	17 (7.3)	139 (59.4)	2.23 ± 0.46	2.077	0.354
Do you handle fish with the wounded hand which are not properly covered?	77 (32.9)	37 (15.8)	120 (51.3)	2.37 ± 0.58	2.887	0.236
Do you properly clean the working area before and after handing out fish?	101 (43.2)	50 (21.4)	83 (35.4)	2.61 ± 0.30	5.529	0.063
Do you wash your hands after handling spoiled or contaminated fish?	33 (14.1)	49 (20.9)	152 (65.0)	2.02 ± 0.66	1.639	0.441
Do you store leftover fish in the refrigerator?	158 (67.5)	0 (0)	76 (32.5)	2.53 ± 0.28	6.318	0.012*
Do you check the shelf life of fish during the delivery time?	121 (51.7)	81 (34.6)	32 (13.7)	2.35 ± 0.35	19.183	0.000*
Total mean score of practices of WFHs				23.08 ± 4.24^b^		
Mean of scores				2.31		

^a^ *n* (%) – number of respondents (percentage of respondents).

^b^ Total (mean ± standard deviation) score of the respondents.

* Statistically significant (*p* < .05).

More than 85% of DFHs showed an interest in eating or drinking in their workplace, while only 14.3% of handlers agreed with smoking in their workplace (Table 7). Only about 38% of DFHs use modern fish dryers for drying fish, while others prefer the traditional sun drying process for its simplicity and versatility, even though the occurrence of dirt and grime, insect infestations, including eggs and larvae, and low final product quality are all typical concerns with sun‐dried items (Nowsad, 2007). According to our findings, <30% of DFHs were concerned about discarding spoiled and contaminated fish from the drying process. Our present finding also correlates with the descriptions of Alam (2005) and Flowra et al. (2013), who also found the use of low‐quality fish for the drying process. Additionally, the undesirable attitudes toward the implementation of food safety strategies are caused mainly by the inadequacy of results‐oriented proper training, relevant time management, inadequate facilities, weak food safety legislation, and poor management of farm managers and owners. It is thus important to teach conventional processes for DFHs to be able to increase product quality through modern techniques. However, the accuracy of these fish handler practices could not be validated because this research did not include the monitoring of fish handlers at their fish processing sites. According to Soon and Baines (2012) and Powell et al. (2011), good food safety knowledge is crucial to preventing foodborne illnesses and enhancing positive attitudes toward food safety knowledge practices.

**TABLE 7:** Food safety and hygiene practices of dry fish handlers (DFHs) (*n* = 258) in Cox's Bazar

Statements	Responses of dry fish handlers^a^ *n* (%)					
	Agree *n* (%)	Neutral *n* (%)	Disagree *n* (%)	Mean ± *SD*	χ^2^	*p*‐Value
Do you wash your hands properly after you end your work for the day?	168 (65.1)	57 (22.1)	33 (12.8)	2.56 ± 0.31	55.586	.000*
Do you use clean hand towels to wipe your hands after washing?	65 (25.2)	104 (40.3)	89 (34.5)	1.88 ± 0.57	217.810	.000*
Do you smoke in your workplace?	37 (14.3)	145 (56.2)	76 (29.5)	1.96 ± 0.65	123.275	.000*
Do you eat or drink in your workplace?	221 (85.6)	10 (3.9)	27 (10.5)	2.75 ± 0.21	17.372	.000*
Do you properly clean the fish drying area before loading new stock?	58 (22.5)	88 (34.1)	112 (43.4)	1.86 ± 0.68	194.003	.000*
Do you inspect the packaging integrity as it is delivered?	83 (32.2)	121 (46.9)	54 (20.9)	2.01 ± 0.52	218.773	.000*
Do you wash wet fish under running clean water before drying process?	106 (41.1)	40 (15.5)	112 (43.4)	2.49 ± 0.36	148.789	.000*
Do you use a modern fish dryer for drying fish?	98 (38.0)	0 (0)	160 (62.0)	2.67 ± 0.24	169.406	.000*
Do you concern about discarding spoiled and contaminated fish from the drying process?	69 (26.7)	75 (29.1)	114 (44.2)	2.46 ± 0.38	235.186	.000*
Do you use any insecticides during fish drying process?	38 (14.7)	81 (31.4)	139 (53.9)	2.14 ± 0.55	160.227	.000*
Total mean score of practices of DFHs				22.78 ± 4.47^b^		
Mean of scores				2.28		

^a^ *n* (%) – number of respondents (percentage of respondents).

^b^ Total (mean ± standard deviation) score of the respondents.

* Statistically significant (*p* < .05).

### 3.6 Correlation among the knowledge, attitudes, and practices of fish handlers

The correlation of KAP of fish handlers is displayed in Table 8. A significant positive correlation was observed between knowledge with attitudes (*r*_s_ = .584 for WFHs and 0.475 for DFHs, *p* < .05), knowledge with practices (*r*_s_ = .472 for WFHs and 0.518 for DFHs, *p* < .05), and attitudes with practice (*r*_s_ = .549 for WFHs and 0.326 for DFHs, *p* < .05). The correlation values indicated a significant association between WFHs' and DFHs' KAP. Other scholars have also shown a strong positive correlation between KAP (Al‐Shabib et al., 2016; Sani & Siow, 2014). Multiple types of research have also suggested that food handling/handler errors are the leading causes of foodborne disease across the world (Osaili et al., 2017; Todd et al., 2009). As a result, the most important measure for foodborne illness preclusion is training on food safety for food handlers (WHO, 2017). Hence, the supervisory members of the fish market and processing facilities must initiate a functional and intensive training program emphasizing food safety and hygiene issues, and suitable documentation ought to be supplied throughout the training program to improve food safety among fish handlers.

**TABLE 8:** Correlation among knowledge, attitudes, and practices level of wet fish handlers (WFHs) and dry fish handlers (DFHs)

Level	Wet fish handlers (WFHs)	Dry fish handlers (DFHs)	*p*‐Value
Knowledge–attitudes	0.584*	0.475*	.00
Knowledge–practices	0.472*	0.518*	.00
Attitudes–practices	0.549*	0.326*	.00

* Correlation is highly significant at the *p* < .001 level.

## 4 CONCLUSIONS AND RECOMMENDATIONS

According to the findings of our study, both wet and dry fish handlers had adequate levels of KAP regarding food safety and hygiene. However, an inadequate knowledge level was observed concerning some aspects, like foodborne disease occurrence, that need to be upgraded. Lack of knowledge in such areas may increase the risk of foodborne disease incidences. Launching a compulsory awareness training program at regular intervals will help the handlers to improve their knowledge level and to process or handle the fish in a safer way to minimize foodborne disease occurrences. Thus, the government and other relevant regulatory authorities should implement strict rules in training, law, and licensing. In addition, a comprehensive, methodical, and efficient organizational approach is needed to ensure adequate food safety procedures. Further research should be conducted so that the scores of fish handlers in the other regions of Bangladesh may be compared to assess the entire scenario of the fish market and processing sites and, thus, compliance with food safety regulations and procedures. Additionally, monitoring of fish handlers' practices at their working sites and physical inspections and checks, such as a microbial load inspection of their hands and working areas, will be performed in the future.

## ACKNOWLEDGEMENT

The authors acknowledge the respondents who took part in this study for their active participation and helpful cooperation during the research work.

## FUNDING INFORMATION

This research did not receive any specific grant from funding agencies in the public, commercial, or not‐for‐profit sectors.

## CONFLICT OF INTEREST

The authors declare that they have no conflict of interest.

## Data Availability

All data generated or analysed during this study are included in this published article.
